# Development of a low pollution medium for the cultivation of lactic acid bacteria

**DOI:** 10.1016/j.heliyon.2023.e22609

**Published:** 2023-11-25

**Authors:** Xóchitl Nochebuena-Pelcastre, Ana Karen Álvarez-Contreras, Marcos Francisco Hernández-Robles, Iván Natividad-Bonifacio, José Carlos Parada-Fabián, Elsa Irma Quiñones-Ramirez, Carlos Ramón Vazquez-Quiñones, Carlos Vázquez Salinas

**Affiliations:** a Departamento de Biotecnología, División de Ciencias Biológicas y de La Salud, UAM Iztapalapa, Av. Sn. Rafael Atlixco 186, Vicentina, CP 09340, Iztapalapa, Mexico; b Escuela Nacional de Ciencias Biológicas, Instituto Politécnico Nacional, Prol. de Carpio y Plan de Ayala S/n, Col. Santo Tomás, 11340, Ciudad de México, Mexico

**Keywords:** Culture media, Lactic acid bacteria, *Lactobacillus*, *L. acidophilus*, *L. plantarum*

## Abstract

Protein rich culture media are employed in the production of lactic acid bacteria (LAB); however, production costs are high. In this work media formulation and evaluation for LAB production were conducted considering physiological properties of lactic acid bacteria. Consumption efficiency (*E*), yield production (*Y*) and specific substrate consumption rate (q_S_) values as response variables were used. Four culture media were used: (1) Man Rogosa Sharp (MRS); (2) cabbage liquor (MC); (3) a new balanced culture medium (MX); and (4) MX supplemented with cabbage liquor (MXC). The culture media were evaluated using two strains: *Lactobacillus acidophilus* ATCC 4356 and *Lactiplantibacillus plantarum* ATCC 10241. The *E*_GLU_ for *L. plantarum* was 100 % in the three media and Y_X/S_ value was 0.02 ± 0.003 in MRS and MX, while Y_LAC/S_ was 0.57 ± 0.03 in MRS and 0.51 ± 0.02 in MX. In MXC, the value obtained for Y_X/S_ was 0.07 ± 0.002 while Y_LAC/S_ was 0.47 ± 0.04. Specific glucose consumption and lactate formation rates for *L. plantarum* in MRS and MX media did not show significant differences. These results suggest that MX and MXC can be used for efficient production of the LAB at low cost.

## 1 Introduction

Lactic acid bacteria (LAB) are highly versatile microorganisms with a wide range of industrial applications [1]. These anaerobic, Gram-positive, and fermentative microorganisms produce lactic acid as the primary secondary metabolite under defined culture conditions, which is an essential component of many industrial products [2,3]. Additionally, LAB are employed in the production of various dairy products such as yogurt, buttermilk, cheese, beverages, and fermented vegetables, which are widely consumed worldwide [4]. LAB are commonly present as a part of the microbiome in humans and can be found in the mouth, intestine, and vagina of mammals [5,6].

LAB can utilize two distinct metabolic pathways: the Embden-Meyerhof-Parnas pathway, which is common in homofermentative bacteria, leading to the production of almost exclusively lactic acid, and the 6-phosphoketolase pathway, which is unique to heterofermentative bacteria, and can result in the production of final products such as ethanol, acetate, and CO_2_ [7,8]. Nevertheless, both pathways can be present in LAB strains under specific culture conditions [9].

Equations eq. 1, eq. 2, eq. 3 illustrate glucose (C_6_H_12_O_6_) oxidation in both pathways. The Gibbs free energy (ΔG°) values are low for both pathways, which limits biomass production due to thermodynamic changes. Thus, the formation of biomass is dependent on ATP production, and in the case of lactic acid fermentation, the production of ATP is limited, resulting in minimal microbial biomass production.

(kJ/mol Gluc)

eq. 1 C_6_H_12_O_6_ + 2Pi + 2ADP^+^ → 2CH_3_CHOHCOOH + 2ATP ΔG° = −30.6

eq. 2 C_6_H_12_O_6_+ 2Pi + 2ADP^+^ → 2CH_3_CH_2_OH+ 2CO_2_ + 2ATP ΔG° = −47.0

eq. 3 C_6_H_12_O_6_+ 2Pi + 2ADP^+^→ CH_3_CHOHCOOH + CH_3_CH_2_OH + CO_2_+2ATP ΔG° = −32.4

Several culture media have been proposed to produce lactic acid and biomass, such as Rogosa Selective *Lactobacillus* Agar (LBS) [10], which is widely used in clinical settings. Its chemical composition includes trypticasein (10 g L-1), yeast extract (5 g L-1), and a solution of salts and glacial acetic acid. Another widely used medium is Man Rogosa Sharpe medium (MRS), which contains meat (10 g L-1) and yeast extract (5 g L-1), as well as peptone (10 g L-1). MRS medium is usually employed to increase LAB viable counts (CFU mL-1); however, these microbial growth quantification results are inappropriate for obtaining mass balances of the process [11]. Despite a lack of quantitative evaluation criteria, the MRS medium is the most widely used for the study and production of LAB [12]. Many studies have shown that the high production cost of LAB is directly related to the nitrogen source, with protein and yeast extract being the most expensive components [13].

There is evidence suggesting that LAB growth can be promoted by using agricultural products as substrates or growth factor sources [14]. For example, the strain *Lactiplantibacillus plantarum* has been used as an inoculum with agricultural sources such as alfalfa, wheat bran, corn, corncob, and wheat soy straw for lactic acid production, with lactic acid concentrations of 44 and 46 g L-1 being obtained with soybean and alfalfa, respectively [15]. However, the process was not characterized in terms of consumption efficiency, yield production, specific rates, and mass balance data.

LAB, including *Lactobacillus acidophilus* ATCC 4356 and *L. plantarum* ATCC 10241, are frequently utilized as probiotics due to their potential health benefits for humans [16,17]. *L. acidophilus* is commonly found in the human gastrointestinal tract and is known for its ability to produce lactic acid, which can lower pH levels and create an unfavorable environment for harmful bacteria [18]. Similarly, *L. plantarum* is known for its probiotic properties, such as its ability to modulate the immune system and enhance gut health [19].

While both L.L. *acidophilus* and *L. plantarum* share some physiological properties, such as their ability to ferment carbohydrates and produce lactic acid, there are also notable differences in their metabolism and characteristics. for example, *L. acidophilus* is a homofermentative bacterium that exclusively produces lactic acid, while *L. plantarum* is a facultative heterofermentative bacterium that can produce other end products like acetic acid, ethanol, and CO_2_ [20].

To fully understand the potential of these probiotics, it is essential to define their consumption efficiency and specific rates. Consumption efficiency refers to the proportion of substrate that is converted into biomass and metabolic end products, while specific rates refer to the rate at which substrates are consumed and products are formed [21]. By determining these properties, we can better understand the behavior of these bacteria in various environments and optimize their use as probiotics or for industrial applications [22].

In Mexico close to 200,000 tons of cabbage are produced every year. An important amount (around 20 %) of this vegetable is rejected as plant waste (or byproduct), therefore, it might be used as a growth factor source for LAB production. The use of this byproduct could diminish industrial cost for production of LAB as probiotic [23]. The purpose of this work was to evaluate the effect of using several culture media which varied in their protein content and the addition or not of cabbage liquor, on the performance of two strains, *Lactobacillus acidophilus* ATTCC 4356 and *Lactiplantibacillus plantarum* ATCC 10241. Variables of response as mass balances, substrate consumption efficiencies, yield and specific rate values were used.

## 2 Materials and methods

### 2.1 Lactic bacteria

*Lactobacillus acidophilus* ATCC 4356 and *Lactiplantibacillus plantarum* ATCC 10241 were used as model strains. These LABs were acquired from the Universidad Nacional Autónoma de México, Facultad de Química.

### 2.2 Culture media

Four culture media were used: (1) Man Rogosa Sharp medium (MRS); (2) cabbage liquor; (3) a new balanced culture medium (MX); and (4) MX supplemented with cabbage liquor (MXC) (Table 1). The C/N ratio for MRS medium is close to two which represents an excess of nitrogen content considering that the C/N ratio for many different biomass productions is around five. It has been determined that the C/N ratio for microbial growth must be twice or higher. Nitrogen content in the yeast and meat extracts was assumed to be 70 % on average. From the empirical consideration (where it is supposed that 10 and 90 % are fated for biomass and lactate production, respectively). In all C/N estimations, glucose was considered as the only source of carbon. The C/N ratio of MX was close to 15. The MXC is like MX but instead of water cabbage liquor was used. Cabbage liquor production was as follows: cabbage leaves were washed with water three times and disinfected with colloidal silver (8 drops per liter of water) and then allowed to dry. In aseptic conditions, 500 g of leaves (wet weight), were passed through a liquor extractor (Olympic, model No. 1000). The resultant liquor was collected and stored at 4 °C. The cabbage liquor was employed as culture medium alone, and it was also used as complement for the MX medium (MXC).

**Table 1:** Composition of MRS, MX broth, and MXC medium with cabbage liquor.

Ingredients g/L	MRS	MX	MXC
Peptone	10.0	3.2	3.2 g
Yeast extract	5.0	0.5	0.5g
Beef extract	10.0	0.2	0.2 g
Glucose	20.0	20	20 g
Dipotassium phosphate	2.0	2.0	2 g
Magnesium sulfate 7H_2_O	0.1	0.2	0.2 g
Manganese sulfate 4H2O	0.05	0.05	0.05 g
triammonium citrate	2.0	2.0	2 g
sodium acetate trihydrate	5.0	5.0	5 g
Polysorbate 80	1.0	–	–
Cabbage liquor	–	–	1.0

### 2.3 Lactic acid bacteria culture

In sterile conditions, lactic acid bacteria culture was conducted in a 3 L working volume instrumented reactor (seed stock). A volume of 1.5 L of cabbage liquor alone, MRS, MX or MXC media were placed in the sterile reactor. Each medium was inoculated with *L. acidophilus* or *L. plantarum* with a final microbial protein concentration of 0.07 ± 0.02 gg LL^−1^. After inoculation, the culture was stirred at 100 rpm. Initial pH was maintained at 6.5 while the temperature was 30 ± 2 ° ^o^C as indicated in other works [24]. The experiments were done in triplicate. For viable count, 5 mL samples were taken from time zero every hour, the LAB were grown in MRS agar culture plates by surface dispersal with 0.1 mL of the culture and incubated at 30 ± 2 ° ^o^C for 72 h. Each sample was centrifuged at 5000×*g* for 15 min, the supernatant was collected in sterile tubes and stored at −20 °C and used for determination of the response variables.

### 2.4 Sample analysis

The gas chromatography samples were prepared using 1.5 mL of supernatant and adding 0.2 mL HCl 5 N. The soluble and insoluble protein content of the cabbage was determined by colorimetric method [25] while its carbohydrate content was analyzed by the sulphuric phenol method [26]. In all cases, the cultures were evaluated by using the following response variables: consumption efficiency (*E*, [Substrate consumed/total substrate consumed] × × 100), yield products (*Y*_X/S_, [C-substrate produced/C-substrate consumed), specific consumption rate (q, [C-substrate/time x C-biomass]) [27] and mass balances.

### 2.5 Analytical methods

The Lowry method was used to measure the soluble and insoluble (microbial biomass) protein. Glucose and lactate were quantified by an enzymatic method using a YSI 2700 equipment (YSI Incorporated) [28] with glucose and lactate membranes (YSI 2365 and 2329, respectively). A lineal response for l-lactate and d-glucose was obtained in the range from 0 to 0.5 gg LL^−1^ and from 0 to 2.5 gg LL^−1^ interval, respectively. In both cases the coefficient of variation was ±2 %. Volatile fatty acids (VFA, acetate, propionate and butyrate) and ethanol were quantified from the supernatant with a Varian Star 3400 gas chromatograph equipped with a flame ionization detector and a Varian 4400 integrator. Nitrogen at a flow of 30 mmLL minmin^−1^ was used as the mobile phase in a 1.2 m long, 1/8” diameter steel column packed with 80/100 mesh (Poropak Q). The following temperatures were set from 180 °C to 235 °C (rate of 27 ° ^o^C/min); injector, 250 °C; detector, 240 °C. A flow of 300 mmLL minmin^−1^ of air and 30 mmLL minmin^−1^ of hydrogen were used. A standard curve using a mixture of VFA and ethanol at concentrations from 100 to 1000 mmgg LL^−1^ for each compound was made. The determination coefficient for each curve was 0.96. The samples were analyzed twice, and the coefficient of variation was negligible (around 4 %).

## 3 Result

The results of *L. acidophilus* in MRS medium are shown in Table 2. Glucose was consumed within 25 h of culture, although the higher portion (90 %) was consumed in the first 12 h. Despite the consumption efficiency value was low; the yield of lactate (Y_LAC/S_) was very high. Thus, the process was homofermentative. From a metabolic point of view, the value of Y_LAC/S_ indicated that glucose was mainly used to obtain energy, 0.06 mol of ATP approximately, if equation (1) is considered. Therefore, a very small biomass production could be expected as Y_X/S_ indicates. During culture of *L. acidophilus,* a decrease close to 26 % in total soluble protein apparently linked to microbial growth was seen. It was calculated that 31 % of glucose added was consumed (0.62 g glucose L^−1^ or 0.25 glucose-C g L^−1^) and used for microbial growth. If 70 and 80 % of the total protein content proceed from yeast and meat extract respectively, the potential protein consumption will correspond to 7.5 gg LL^−1^ (1.2 gg NN L^−1^ considering the nitrogen alone).

**Table 2:** Response variables determined by kinetics in MRS commercial medium and the formulated media MX and MXC with *Lactobacillus acidophilus* and *Lactiplantibacillus plantarum*.

Culture medium	Strains	Y _x/s_(g C biomass/g C consumed)	Y _lac/s_(g C lactate/g C consumed)	⎩_glu_	Final CFU/mL (X10^8^)
MRS	*Lactobacillus acidophilus* ATCC 4356	0.14	0.92	30.8	1
MRS	*Lactiplantibacillus plantarum* ATCC 10241	0.02	0.57	100	2
MX	*Lactiplantibacillus plantarum ATCC 10241*	0.02	0.51	100	2
MXC	*Lactiplantibacillus plantarum ATCC 10241*	0.07	0.47	100	25

**Fig. 1:**
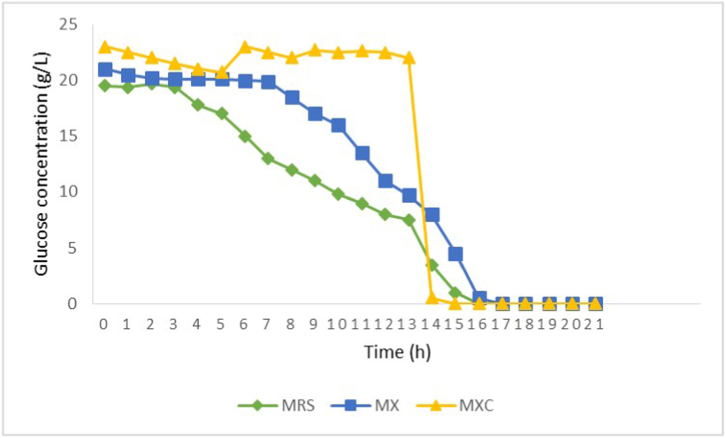
Glucose consumption in *L. plantarum* ATCC 10241 using the culture media MRS (green line), MX (blue line) and MXC (yellow line). The bacterial culture was incubated at 37 °C/24h. The faster decrease in glucose consumption observed in MXC and MX medium compared to MRS medium could be attributed to factors such as carbon source availability, nutrient composition, and differences in metabolic pathways.

**Fig. 2:**
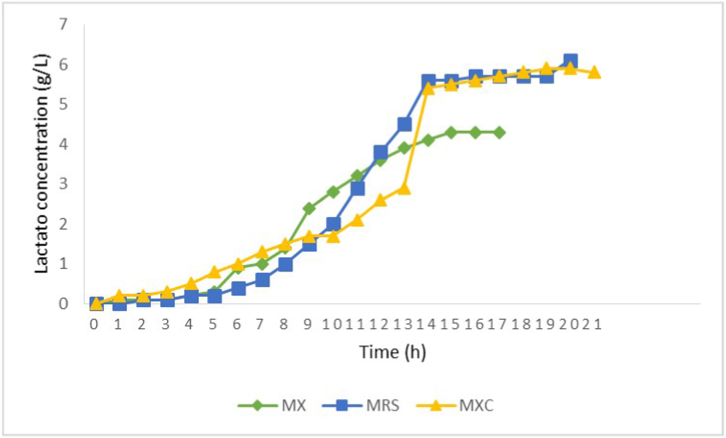
Lactic acid production of *L. plantarum* ATCC 10241 on MRS (blue line), MX (green line) y MXC (yellow line) media. The bacterial culture was incubated at 37 °C/24h, lactate concentrations were determined at 1 hone hour intervals. The MX and MXC media demonstrated similar and higher lactate production compared to the MRS medium. The presence of specific compounds in the MX and MXC media played a crucial role in promoting lactic acid production by *L. plantarum* ATCC 10241, offering valuable insights for optimizing fermentation conditions and improving the efficiency of lactic acid production processes.

Fig. 1, Fig. 2 shows *L. plantarum* strain behavior in MRS, MX and MXC medium. It can be observed that lactate fermentation followed the normal pattern where glucose is firstly consumed and then lactate is accumulated in culture medium. The *E*_GLU_ was 100 % in all cases and ethanol and acetate were detected. Comparing L.L. *plantarum* with *L. acidophilus*, the *E*_GLU_ values for the former were lower. A mass balance based on yield values for glucose consumption in MRS medium is shown in equation (4):

eq. 4 Y_total_ = Y_lac/s_ + Y_x/s_ + Y_eta/s_ + Y_ace/s_ = 0.57 + 0.02 + 0.34 + 0.03 = 0.96

From these values the fermentation profile of *L. plantarum* was heterofermentative as Y_LAC/S_ and Y_ETA/S_ were the highest values. In fact, it is known that *L. plantarum* is a typical homofermentative strain in MRS.

This behavior could be related to pH changes from 6.5 to 3.0. These authors determined that pH is a very important factor for controlling the lactic fermentation profile. These results clearly show that for *L. acidophilus* culture the protein amount added was in excess. The MX was formulated assuming empiric considerations in which the production of biomass in a fermentative process uses 10 % of the added glucose. If 20 gg LL^−1^ of glucose (0.8 gg CC L^−1^ from 2 gg LL^−1^) are present, 1.6 g biomass L^−1^ might be produced and 1.2 g protein L^−1^ should be necessary (considering that 12 % of the total weight correspond to nitrogen, namely, 0.19 gg NN L^−1^ it should correspond to). Thus, the addition of 3 gg LL^−1^ of protein to the MX would be in excess. Nevertheless, this concentration is almost 10 times lower than that in MRS medium (around 29 gg LL^−1^). Moreover, as meat and yeast extracts were considered as growth factors, their concentrations were reduced in 90 and 98 %. Because L.L. *plantarum* showed better results according to the response variables chosen, this strain was used to assess the MX and MXC. The results are shown in Fig. 1, Fig. 2. After 17 h of culture, glucose consumption in MX and MXC was as high as in the MRS medium. In MX, 75 % protein was apparently consumed, and microbial growth was very low, probably because of the hydrolytic activity of the culture, as mentioned before. Despite the protein concentration being significantly lower, *E*_GLU_ was 100 %, Y_LAC/S_ was 0.51 and Y_X/S_ was 0.02, like the results obtained in MRS. A C/N ratio of 17 in MX was obtained, which can be considered adequate for promoting microbial growth, however, growth will be stringed by ATP limitation. The results suggest that MX can be used instead of MRS. Moreover, the medium culture and the effluent treatment for nitrogen elimination costs will be lower. When L.L. *plantarum* was grown in MRS medium, the pH value diminished from 6.5 to 3.4 whereas *E*_GLU_ and lactate production also diminished.

Table 3 shows the specific rates values (q_GLU_ and q_LAC_) for both lactic strains in each culture media (MRS, MX and MXC). As specific growth rate values were negligible, they were not included. The process might be represented by

eq. 5 q_s_ = μ/Y_X/S_ + q_P_/Y_P/S_

Where q_S_ is the substrate consumption rate, μ is the specific growth rate, q_P_ (or q_LAC_) is specific production rate and Y the yields for biomass and product (lactate) formation. equation (5) is derived from a mass balance of the limiting substrate (glucose, energy, and carbon source).

eq. 6 dS/dt = dS_X_/dt + dS_P_/dt

**Table 3:** Specific rates for lactate production (q_lac_) and glucose consumption (q_glu_), in MRS, MX y MXC media inoculated with *Lactobacillus acidophilus* and *Lactiplantibacillus plantarum*.

Medium/species	q_glu_ (g glucose/g biomass^.^h)	q_lac_ (g lactate/g biomass^.^h)
MRS/*Lactobacillus acidophilus*	2.15	2.88
MRS/*Lactiplantibacillus plantarum*	1.4	1.1
MX/*Lactiplantibacillus plantarum*	1.8	1.6
MXC/*Lactiplantibacillus plantarum*	ND	0.95

This equation is empirically resolved and simplified by Meza-Escalante et al. [29] indicating that the first term of eq, 4 is almost null resulting in eq. (6).

eq. 7 q_S_ = q_P_/Y_P/S_

This equation shows that lactic acid fermentation is a dissimilative process, where the product formation is not dependent on the microbial growth, or it is negligible. Thus, in the lactic fermentation process the big amounts of protein are unnecessary. The Monod equation can be used to predict the behavior of the lactic fermentation (eq. (7))

eq. 8 q_S_ = q_MAX_[S]/K_S_ + [S]

Where q_MAX_ is maximum specific rate and K_S_ the substrate affinity constant. This equation is evidence that the rate process is dependent on the substrate concentration and the K_s_ value.

## 4 Discussion

Chemical analysis of cabbage revealed that it contains 2.04 ± 0.05 g of protein and 0.19 ± 0.07 mg of total sugars per 100 g of wet weight. However, a study by Ref. [30] reported lower levels of protein (1.47 g) and higher levels of sugar (5.71 g) per 100 g of dry weight cabbage (see Table 4). The discrepancy in sugar content may be attributed to various factors, such as the physiological state of the plant, environmental conditions or storage, or the inclusion of total fibers in the sugar measurement. When L.L. *acidophilus* and *L. plantarum* cultures were evaluated using cabbage liquor as culture medium, no changes were observed neither for microbial protein concentration nor for lactate production after 24 h of culture. This revealed that the substrate concentrations in cabbage liquor were not enough for LAB production. Based on empirical considerations, it is estimated that in a fermentative process, 10 % of carbon glucose is used for biomass formation. If we assume that 50 % of the biomass is carbon and that 0.5 g of biomass is produced per liter of consumed glucose, then only 0.06 g L^−1^ of glucose is required for biomass formation. It is worth mentioning that, in MXC medium, glucose was used hard for approximately 13 h cultivation, and 14 h after, glucose was drastically decreased (Fig. 1). Microorganisms often switch their metabolic strategies depending on the availability of nutrients [31]. It is possible that, at the 14th hour, the microbial population entered a different growth phase or switched to an alternative carbon source, which led to a reduced reliance on glucose. It has been reported that this metabolic adaptation could be a response to changing environmental conditions, depletion of glucose, or the accumulation of by-products that triggered the shift [32]. Therefore, the decrease in protein observed in this study cannot be attributed to microbial growth, as it would require only a minimal amount of glucose to produce biomass. Instead, the decrease in protein content may be due to hydrolysis phenomena, as reported by Fraberger et al. [33], which could not be detected by the method used in our study.

**Table 4:** Chemical composition per 100 g wet weight of cabbage (Uuh-Narvaez & Segura-Campos, 2021).

Water	93 %
Carbohydrates	5.71 g
Proteins	1.43 g
Lipids	Traces
Calcium	47.14 mg
Phosphorus	22.86 mg
Iron	0.57 mg
Potassium	245.71 mg
Sodium	18.57 mg
Vitamin A	128.57 IU
Thiamine	0.06 mg
Riboflavin	0.03 mg
Niacin	0.29 mg
Ascorbic acid	47.14 mg
Energy value	21.43 cal

In addition to thermodynamic considerations, the limited biomass production observed may also be attributed to the C/N ratio of the medium [34]. Studies have shown that a C/N ratio as low as 2 in the culture medium can lead to an unbalanced medium and hinder biomass production [35]. It is also crucial to maintain appropriate pH levels during the fermentation process. Liu et al. [36] reported that acidic pH levels favor the formation of lactate dehydrogenase enzymes, which leads to increased lactic acid production and decreased biomass generation. Therefore, pH control is a critical factor in optimizing fermentation conditions for maximum biomass production [37]. It is important to note that the specific rates of *L. acidophilus* and *L. plantarum* in the MRS medium (Table 3) vary significantly, while the specific rates in MRS and MX for *L. plantarum* are comparable: *L. acidophilus*, being a homofermentative organism, has a more specialized metabolic capacity for the production of lactic acid as the primary end product during carbohydrate fermentation. This means that its consumption of substrates like glucose can be faster and more efficient compared to *L. plantarum* [38]. On the other hand, *L. plantarum*, as a heterofermentative organism, has a more diverse metabolic pathway, allowing it to generate a variety of end products during fermentation, including lactic acid, carbon dioxide, ethanol, and acetic acid [39]. This implies that the consumption of glucose by *L. plantarum* could be slower compared to *L. acidophilus*, as energy and resources are distributed among different end products (Table 2, Table 3).

This suggests that the fermentation process in MX is equally efficient and productive as in MRS, not just in terms of yield and efficiency, but also in specific rate, despite the vast difference in cost between the two media. The growth kinetics of *L. plantarum* were assessed using MX and MRS media under the same culture conditions. Interestingly, both media yielded a final cell concentration of 2 × 10^8^ CFU mL-1, indicating that MX can be as effective as MRS for bacterial growth. However, relying solely on viable count as a criterion for evaluating fermentation processes may not be entirely reliable. EGLU, YX/S, and YLAC are other important parameters that can provide valuable information about fermentation efficiency and yield [40]. It's worth noting that the cell concentration obtained using MXC was much higher, reaching 25 × 10^8^ CFU mL-1, possibly due to growth factors present in cabbage [41].

Considering that during lactic fermentation, the microbial biomass production is always low as the biomass formation is limited due to the thermodynamic restriction, it is evident that the nitrogen content required (as protein concentration) in the SL and MRS media must be low [42,43]. In this sense, the use of MRS medium as well as its modifications for the improvement of viable count values have been reported. In these studies, the factors modified included pH of the culture, temperature incubation, carbon source and the addition of inhibitors [44,45], but no modification on protein content has been made. It is reported that Barney-Miller-Brewery medium (BMB) which uses tomato juice amongst other substances produce better results than MRS, although the sole evaluation criteria were *Lactobacillus* spp. and *Pediococcus* spp viable count. Polak-Berecka et al. [46] using *Lactobacillus rhamnosus*, developed a medium containing all L-aminoacids, 13.4 gg LL^−1^ of glucose and a mixture of vitamins. They reported 5.183 gg LL^−1^ of biomass. Despite the yield value obtained was high, the culture medium preparation is complex and expensive.

In general, culture media are assessed by means of LAB viable count [47], but response variables such as consumption efficiency, yield product (Y), specific consumption rate (q) and mass balance have not been determined for evaluating the media performance [48]. The omission or lacking determination of these variables does not allow a precise evaluation of culture and the process. Nevertheless, if mass balance is performed, it is possible to estimate that microorganisms use less than 5 % of the protein added to the culture. Thus, although a large amount of protein is added, scanty biomass will always be produced due to restriction in the ATP production. Therefore, an important protein concentration will remain useless at the end of culture. In fact, the cost treatment of the effluents from LAB culture production might be as expensive as the self-fermentation process [49]. Likewise, the use of high protein concentrations during industrial LAB production reduces the C/N ratio and consequently bacterial metabolism may be modified [50]. In this study, four different culture media were tested for their effectiveness in supporting the growth of *L. acidophilus* and *L. plantarum*. The widely used MRS medium was compared to the MX and MXC media, which were specifically developed for this research. Additionally, a medium made from cabbage must was also tested. However, the results showed that the protein nitrogen-rich MRS medium led to an unpredictable culture of *L. acidophilus*, with significant variation in efficiency and yield. Furthermore, the cabbage must medium produced poor results and was deemed unsuitable for LAB production, although it may still have potential as a source of growth factors. Therefore, the study highlights the importance of formulating and testing specialized culture media for optimizing LAB growth and productivity. The evaluation of *L. plantarum* in MRS, MX, and MXC culture media demonstrated that MX and MXC are viable options for LAB culture, as there were no significant differences observed in their response variables such as efficiency, yield, and specific velocity. Moreover, the formulation and preparation of MX and MXC showed significant cost advantages compared to MRS. Therefore, MX and MXC can be considered as efficient and cost-effective alternatives to MRS for LAB cultivation. These findings highlight the importance of developing and optimizing culture media to enhance the efficiency and reduce the costs of LAB production, which can have significant implications for the industrial production of LAB-based products [51].

The objective of developing culture media is to cultivate LAB efficiently. The findings of this study indicate that it is feasible to create culture media that are eco-friendly and economical. Nevertheless, it was observed that the dissimilatory respiratory process predominated during the growth of LAB, implying that further investigation is required to enhance the formulation of MX and MXC media to yield more probiotic biomass. By continuing to optimize the composition of these media, it may be possible to achieve even better outcomes in terms of LAB production, ultimately leading to the development of more sustainable and cost-effective fermentation processes. Furthermore, the use of cabbage must as a culture medium has additional environmental benefits beyond its low cost and nutrient-rich properties [52]. As a waste material, the use of cabbage must for LAB production can contribute to reducing the environmental impact of food waste, particularly in the context of the circular economy. This approach can also help to promote sustainable food production and reduce the reliance on synthetic culture media, which can be costly and have a significant environmental footprint. However, the optimization of the cabbage must culture medium requires further research and development to fully harness its potential as a sustainable and low-cost alternative for LAB production. Additionally, the adoption of circular economy models in the food industry can create new economic opportunities and contribute to the development of a more sustainable and resilient food system. In addition to the findings on MX and MXC media, this study sheds light on the importance of optimizing culture media for LAB production. By understanding the optimal composition and conditions of culture media, it may be possible to improve the performance and yield of LAB-based products. This could have significant implications for the food industry, as LABs are widely used in the production of fermented foods and beverages, as well as in probiotics and other health supplements. The study also emphasizes the need for sustainable and low-cost alternatives for culture media, as demonstrated by the potential of cabbage must. By utilizing waste materials and promoting circular economy models, the food industry can move towards more sustainable and environmentally friendly practices. Overall, this research highlights the importance of continued exploration and development of culture media for LAB production, with the potential to benefit both the industry and the environment.

## Additional information

No additional information is available for this paper.

## CRediT authorship contribution statement

**Xóchitl Nochebuena-Pelcastre:** Investigation, Methodology, Writing – original draft, Writing – review & editing. **Ana Karen Álvarez-Contreras:** Data curation, Formal analysis, Writing – original draft, Writing – review & editing. **Marcos Francisco Hernández-Robles:** Investigation, Methodology, Writing – original draft, Writing – review & editing. **Iván Natividad-Bonifacio:** Data curation, Formal analysis, Writing – original draft, Writing – review & editing. **José Carlos Parada-Fabián:** Data curation, Formal analysis, Writing – original draft, Writing – review & editing. **Elsa Irma Quiñones-Ramirez:** Conceptualization, Funding acquisition, Resources, Writing – original draft, Writing – review & editing. **Carlos Ramón Vazquez-Quiñones:** Investigation, Methodology, Writing – original draft, Writing – review & editing. **Carlos Vázquez Salinas:** Conceptualization, Funding acquisition, Resources, Writing – original draft, Writing – review & editing.

## Declaration of competing interest

The authors declare that they have no known competing financial interests or personal relationships that could have appeared to influence the work reported in this paper.
